# Comparative Analysis of Transcriptomes Reveals Pathways and Verifies Candidate Genes for Clubroot Resistance in *Brassica oleracea*

**DOI:** 10.3390/ijms25179189

**Published:** 2024-08-24

**Authors:** Fuquan Ce, Jiaqin Mei, Yu Zhao, Qinfei Li, Xuesong Ren, Hongyuan Song, Wei Qian, Jun Si

**Affiliations:** 1 College of Horticulture and Landscape Architecture, Southwest University, Chongqing 400716, China; cfq0419@email.swu.edu.cn (F.C.); zhaoyu@163.com (Y.Z.); feifei1984998@126.com (Q.L.); rxsxy@163.com (X.R.); yuahs@163.com (H.S.); 2 College of Agronomy and Biotechnology, Southwest University, Chongqing 400716, China; jiaqinmay@163.com; 3 Key Laboratory of Agricultural Biosafety and Green Production of Upper Yangtze River, Ministry of Education, Chongqing 400716, China; 4 Academy of Agricultural Sciences, Southwest University, Chongqing 400716, China; 5 Academy of Agricultural Sciences, State Cultivation Base of Crop Stress Biology for Southern Mountainous Land, Southwest University, Chongqing 400716, China; 6 Chongqing Key Laboratory of Olericulture, Chongqing 400716, China

**Keywords:** clubroot, *Brassica oleracea*, transcriptome, resistance mechanism, candidate genes, gene function analysis

## Abstract

Clubroot, a soil-borne disease caused by *Plasmodiophora brassicae*, is one of the most destructive diseases of *Brassica oleracea* all over the world. However, the mechanism of clubroot resistance remains unclear. In this research, transcriptome sequencing was conducted on root samples from both resistant (R) and susceptible (S) *B. oleracea* plants infected by *P. brassicae*. Then the comparative analysis was carried out between the R and S samples at different time points during the infection stages to reveal clubroot resistance related pathways and candidate genes. Compared with 0 days after inoculation, a total of 4991 differential expressed genes were detected from the S pool, while only 2133 were found from the R pool. Gene function enrichment analysis found that the effector-triggered immunity played a major role in the R pool, while the pathogen-associated molecular pattern triggered immune response was stronger in the S pool. Simultaneously, candidate genes were identified through weighted gene co-expression network analysis, with *Bol010786* (*CNGC13*) and *Bol017921* (*SD2-5*) showing potential for conferring resistance to clubroot. The findings of this research provide valuable insights into the molecular mechanisms underlying clubroot resistance and present new avenues for further research aimed at enhancing the clubroot resistance of *B. oleracea* through breeding.

## 1. Introduction

Clubroot is a soil-borne disease caused by *Plasmodiophora brassicae*, an obligate biotrophic protist that specifically infects cruciferous families, including Chinese cabbage, cabbage, radish, cauliflower, and mustard greens [1]. Globally, average yield losses caused by clubroot are estimated to range from 10% to 15%, but under optimal conditions for this pathogen, these losses can escalate to between 30% and 100% [2]. The life cycle of *P. brassicae* is divided into root hair infection and cortical infection [3,4]. During the root hair infestation stage, resting spores sense the host plant, enter root hairs to form primary zoospores, parasitize the surface of the host root, and form primary plasmodium. Then, the plasmodium grows into secondary zoosporangia to form secondary zoospores sporangia. During the cortical infection stage, secondary zoospores are released into the soil directly or through root hairs, where they re-infect root cortical cells or develop into secondary plasmodium. This causes the host plant to produce many dormant spores and swollen roots [5,6]. Once a field is contaminated, *P. brassicae* spores can survive for many years, making the disease difficult to control [7]. Therefore, it is essential to study the molecular basis of clubroot contamination of cruciferous plants.

Plants use physical barriers (such as cell walls, cuticles, waxy layers, and lignin) or chemical barriers (such as phenols, saponins, and mustard oils) to fight pathogens [8]. If these defenses are disrupted, plants activate their defensive immune system, which consists of the pathogen-associated molecular pattern (PAMP)-triggered immunity (PTI) and effector-triggered immunity (ETI) [9,10]. PTIs are the basal immune response of plants and can be suppressed by effectors secreted by pathogens. At the same time, the effector can be recognized by nucleotide-binding leucine-rich repeat (NB-LRR) proteins in plants, leading to a more dramatic immune response to ETI. In recent years, evidence has shown that the interplay between ETI and PTI leads to a more robust defense response against pathogen invasion [11,12]. Although many clubroot resistance (CR) loci have been identified in cruciferae, only three NB-LRR genes, i.e., *CRa* [13], *Crr1a* [14], and *CRb* [15], were cloned mainly from *Brassica rapa*. Moreover, the detailed defense response mechanisms of CR genes remain to be elucidated.

In recent years, comparative transcriptome analyses have focused on the molecular mechanisms of CR in *Brassica* species, revealing that these mechanisms may involve cell wall synthesis, phytoalexin synthesis, salicylic acid (SA) signal transduction, auxin and cytokinin (CTK) syntheses, jasmonic acid (JA) and ethylene signaling metabolism, chitinase synthesis, Ca^2+^ signaling, reactive oxygen species gene activation, phenylpropane pathway [1,16,17,18,19,20], whereas the mechanism of CR in *B. oleracea* remains unclear due to the lack of resistant resource within this species.

To investigate the defense mechanism of *B. oleracea* against clubroot, RNA-seq was applied in this study to two *B. oleracea* pools, which exhibited different resistance levels to *P. brassicae* [21,22]. Comparative analysis of differentially expressed genes (DEGs) was carried out between the resistant (R) and susceptible (S) pools at different time points during the infection, and functional enrichment analysis was applied to understand the resistance-related pathways and genes. The weighted gene co-expression network analysis (WGCNA) was further carried out to drag the hub genes in each important resistance-related pathway. Following this, an initial validation of the gene’s functionality was conducted. This study provides novel insights into the molecular mechanism of CR breeding and the significance of its application in *B. oleracea*.

## 2. Results

### 2.1. RNA Sequencing and Data Analysis

After filtering the raw data (NCBI sra bioproject ID: PRJNA735118), a total of 176.98 Gb of 150 bp paired-end clean reads were obtained from the 24 samples. Each sample had an average of 6.06 Gb of high-quality reads (Supplementary Table S2). The GC content of the high-quality reads across all 24 samples ranged from 46.92% to 47.56%, and the Q30 (reads with an average quality score ≥ 30) was consistently above 93.12%, demonstrating that the sequencing data was of high quality and suitable for further analysis. In each sample, approximately 72.56% to 74.23% of high-quality reads were successfully aligned to the *B. oleracea* reference genome. Additionally, a total of 5105 novel genes were discovered in previously unannotated transcribed regions. Principal component analysis (PCA) showed a high correlation in transcriptome characteristics among the three biological replicates at each time point for both the R and S pools (Supplementary Figure S1). The Pearson correlation coefficients were high (R^2^ > 0.90, in most cases) among the three biological replicates (Supplementary Figure S1), reflecting the high quality and consistency of the RNA-seq data.

### 2.2. Overview of the Transcriptome Profiles in R and S Pool

**Figure 1:**
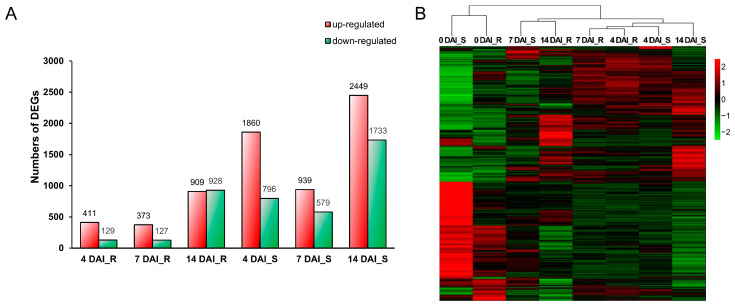
Transcriptomic response profiling in *B. oleracea* roots following inoculation with *P. brassicae*. (**A**) Histogram of number of up- or down-regulated DEGs. The red box represents the number of up-regulated genes; the green box represents the number of down-regulated genes. (**B**) Heatmap of all DEGs of R and S pools at different infection stages compared to 0 DAI. DAI, days after inoculation. R, resistant pool. S, susceptible pool.

To investigate the response of *B. oleracea* to *P. brassicae* at different infection stages, DEGs were identified by comparing the libraries at 4, 7, and 14 days after inoculation (DAI), with the baseline at 0 DAI. A total of 4991 DEGs were detected from the S pool compared to 0 DAI, while 6 DEGs were found from the R pool (Figure 1A). Among them, 2656 (1860 up- and 796 down-regulated), 1518 (939 up- and 579 down-regulated) and 4182 (2449 up- and 1733 down-regulated) DEGs were obtained at 4, 7, and 14 DAI, respectively, compared to 0 DAI in S pool, while relatively few DEGs were obtained at the same point in R pool, 540 (411 up- and 129 down-regulated), 500 (373 up- and 127 down-regulated), and 1837 (909 up- and 928 down-regulated), respectively (Figure 1B). The FPKM values and functional annotations of all DEGs were presented in Supplementary Table S3. To validate the RNA sequencing data, nine DEGs were randomly selected for qRT-PCR analysis. The expression trends of these genes determined by qRT-PCR were consistent with the FPKM values of transcriptome data at all infection stages, confirming the reliability of the RNA sequencing results (Supplementary Figure S2).

To further investigate the differences in CR between R and S pools, we analyzed the DEGs that displayed varied expression patterns in the two pools at different infection stages. These DEGs include resistance genes (R gene) related to ETI, pattern recognition receptors (PRRs) involved in PTI, genes related to cell wall integrity, plant hormone signal transduction, Ca^2+^ influx and respiratory burst oxidase homolog (RBOH), MAPK cascades, transcription factors, and chitinase (Supplementary Table S4). The heatmaps presented in Supplementary Figure S3 reveal a pronounced up-regulation of R genes in the R pool. Conversely, most PTI-related genes, along with genes associated with plant hormone signaling and cell wall structure, were predominantly up-regulated in the S pool.

### 2.3. DEGs Analysis of the R and S Pools at Different Infection Stage

A total of 6016 DEGs were identified from the comparative transcriptomic analysis between R and S pools, using 0 DAI as a baseline. These DEGs were used to construct a heatmap (Figure 1B), which illustrated a similar transcriptomic profile in both pools prior to inoculation. After inoculation, distinct differences in transcriptional responses emerged at various infection stages, highlighting the varied responses of the R and S pools to *P. brassicae* infection.

**Figure 2:**
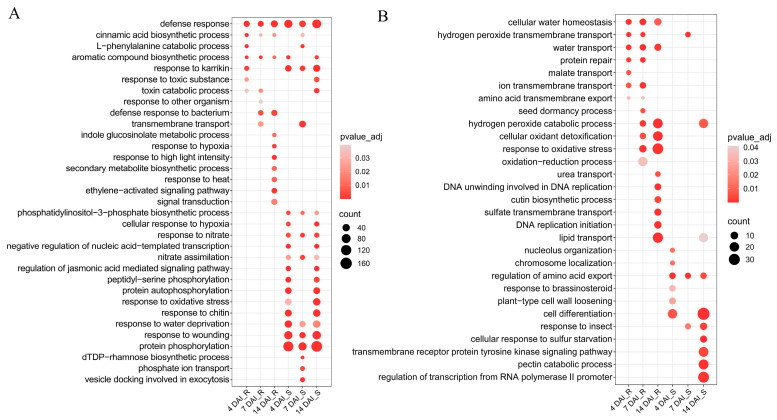
GO enrichment analysis of the differentially expressed genes in R and S pools at different infection stages compared to 0 DAI. (**A**) The GO significant enrichment analysis of up-regulated genes. (**B**) The GO significant enrichment analysis of down-regulated genes. The *y*-axis corresponds to the enriched GO terms, while the *x*-axis represents the varying time points post-inoculation for both R and susceptible S pools. The size of each dot indicates the number of genes enriched for each term, and the color of each dot signifies the adjusted *p*-value, representing the significance of each term. DAI, days after inoculation. R, resistant pool. S, susceptible pool.

To elucidate the biological mechanisms underlying CR, GO enrichment analysis was performed separately on up-regulated and down-regulated DEGs in the R and S pools at different infection stages (Supplementary Table S5). The top 10 significantly enriched biological processes in each pool are displayed in Figure 2. It was found that the progress “defense response to bacteria” at 7 and 14 DAI, “indole glucosinolate metabolic process”, “response to hypoxia”, “signal transduction”, “ethylene-activated signaling pathway”, “response to heat”, “secondary metabolite biosynthetic process”, and “response to high light intensity” at 14 DAI were uniquely enriched in the up-regulated DEGs of the R pool (Figure 2A). Analysis of the DEGs of these progresses found that the 30 DEGs in “defense response to bacteria” included six R genes and seven cysteine-rich receptor-like protein kinase (RLK) genes. Compared to the S pool, the R pool showed induced expression of these DEGs at 7 and 14 DAI. Notably, *Bol013570* exhibited an up-regulated trend in the R pool but a down-regulated trend in the S pool. Similarly, the 28 DEGs in the “signal transduction” process at 14 DAI, which included 20 R genes and three receptor-like protein (RLP) genes, also showed induced expression in the R pool at different infection stages. Down-regulated DEGs were identified to be significantly enriched in 15 biological processes specific to the R pool (Figure 2B). These processes were critical for maintaining cellular homeostasis, responding to environmental stress, and supporting essential life activities.

Upon analyzing processes related to environmental stress response, we found that the group of 30 DEGs categorized under “response to oxidative stress” consisted of 23 peroxidase genes. Additionally, the 23 DEGs categorized under “cellular oxidant detoxification” were all peroxidase genes. This GO enrichment analysis implied that the R pool exhibited robust signal transduction and elicitation of R gene expression, whereas peroxidase genes were down-regulated after inoculation.

In the S pool, the up-regulated DEGs were significantly enriched in 16 biological processes related to cellular signaling, response to environmental stimuli, and metabolic regulation (Figure 2A). Notably, within the “regulation of jasmonic acid-mediated signaling pathway”, 12 out of 16 DEGs were jasmonate ZIM-domain (JAZ) genes. Similarly, the “response to wounding” category included 12 JAZ genes. These JAZ genes were up-regulated in the S pool compared to the R pool. The “response to oxidative stress” process had 21 peroxidase genes that were up-regulated among the 55 DEGs in the S pool, which contrasted with the down-regulation of peroxidase genes in the R pool. The DEGs that were down-regulated were found to be significantly enriched in 11 biological processes that were specific to the S pool (Figure 2B). These genes played crucial roles in plant cellular organization and differentiation, regulation of metabolic processes, and response to environmental stimuli. This analysis revealed that the S pool showed a strong affinity for chitin, with a significant up-regulation of JAZ and peroxidase genes. Conversely, genes associated with cellular differentiation and response to environmental stimuli were down-regulated in the S pool.

**Figure 3:**
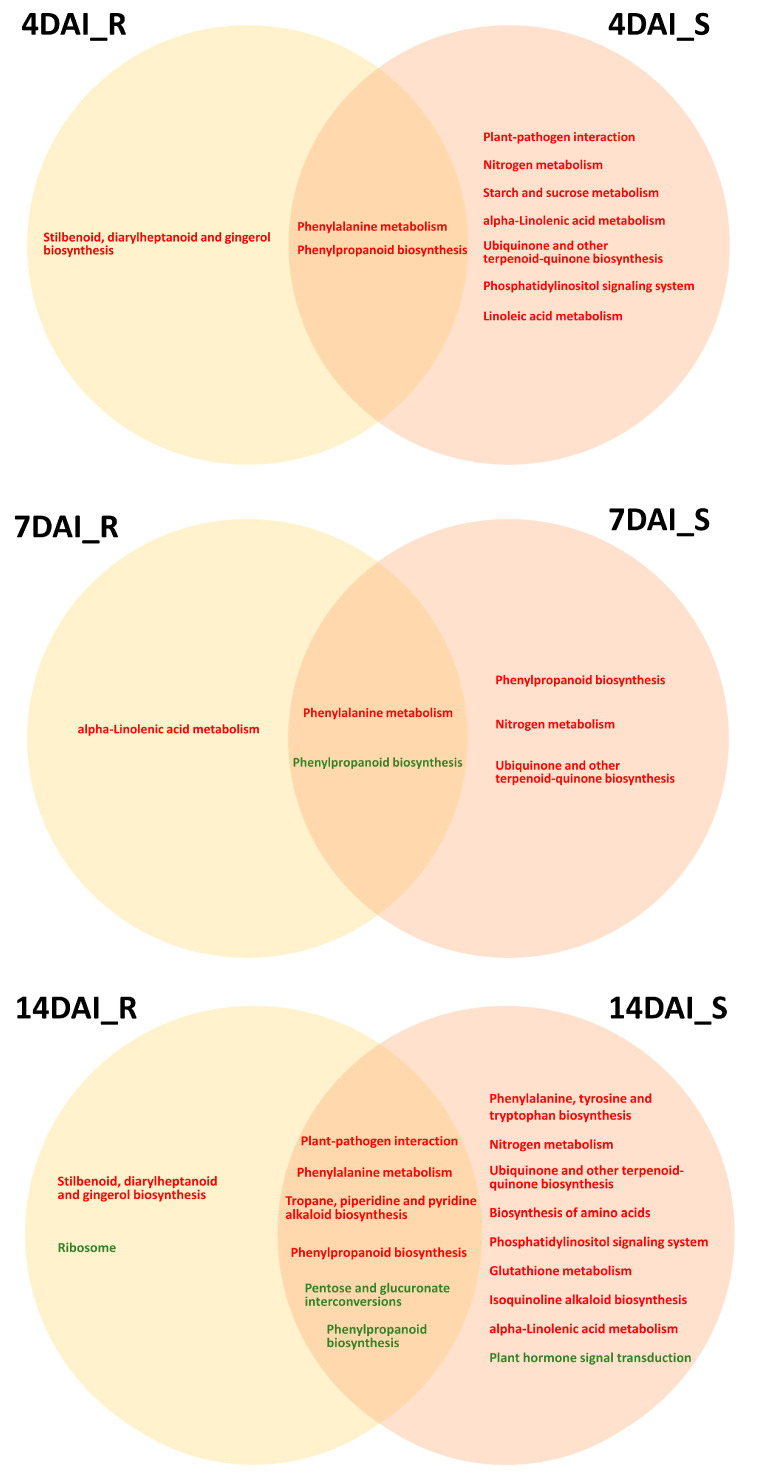
Significant enrichment of up-regulated (in red) and down-regulated (in green) DEGs in KEGG pathways across both pools. DAI, days after inoculation. R, resistant pool. S, susceptible pool.

Furthermore, KEGG enrichment analysis was performed on the up-regulated and down-regulated DEGs in both the R pool and S pool at different infection stages, with a corrected *p*-value < 0.05 (Figure 3 and Supplementary Table S6). The analysis of the significantly enriched KEGG pathways revealed a greater number of enriched pathways for up-regulated DEGs in the S pool compared to the R pool (Figure 3). Notably, the “plant–pathogen interaction” pathway was significantly enriched in the S pool at 4DAI (Figure 3).

Upon integrating the results with GO enrichment analysis, it appears that the S pool’s recognition of *P. brassicae* chitin during the root-hair infection stage leads to significant overall production changes. Conversely, the disease resistance of the R pool was mainly concentrated at the cortical infection stage. The pathways that were enriched in up-regulated DEGs in the S pool, including “nitrogen metabolism” and “ubiquinone and another terpenoid–quinone biosynthesis”, were consistently enriched across all three infection stages (Figure 3). Notably, genes encoding glutamate dehydrogenase, high-affinity nitrate transporter, nitrate reductase, beta carbonic anhydrase 3-like, acyl-activating enzyme, AMP-binding enzyme, and aminotransferase class I and II were up-regulated in the S pool compared to R pool. The down-regulated DEGs in the S pool were found to be specifically enriched in “plant hormone signal transduction” at 14 DAI (Figure 3), with a majority associated with auxin and cytokinin signal transduction. Additionally, up-regulated DEGs in the R pool at 4 and 14 DAI were specifically enriched in the “stilbenoid, diarylheptanoid, and gingerol biosynthesis” pathway (Figure 3), with seven out of eight DEGs encoding Cytochrome P450 (CYP) showing increased expression in the R pool compared to the S pool.

Overall, these findings illuminate the distinct defense strategies and molecular mechanisms employed by the R and S pools in response to *P. brassicae* infection.

### 2.4. Co-Expression Network Analysis and Screening of Candidate CR Genes in B. oleracea

To obtain insight into the molecular mechanisms involved in disease resistance, 6504 valid genes were selected for WGCNA. This analysis identified 14 distinct modules, each representing clusters of highly interconnected genes with similar expression patterns (Supplementary Figure S4A). The Magenta module, with 939 DEGs (855 annotated), was the largest, while the Darkslateblue module, containing 36 DEGs (31 annotated), was the smallest. The Grey module included genes not assigned to other modules. Eight modules—Black, Dark Turquoise, Tan, Brown, Red, Darkslateblue, Darkmagenta, Orangered4, and Lightcyan—were highly correlated with either the R or S pool (|correlation coefficient| > 0.8) (Supplementary Figure S4B). Notably, some modules showed highly correlated expression patterns during specific infection stages in the R pool but not in the S pool, suggesting a potential link to clubroot resistance (CR).

**Figure 4:**
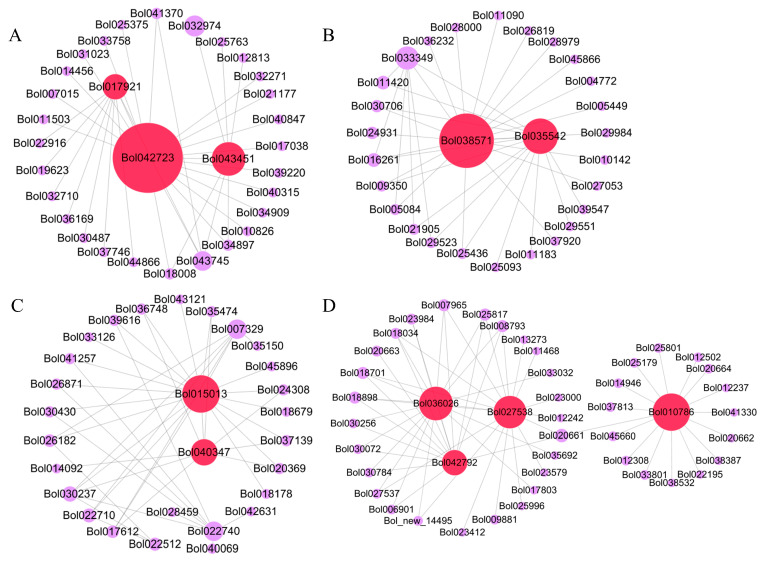
The hub genes identified by the gene co-expression network. The network of “Red” module (**A**), “Black” module (**B**), “Brown” module (**C**), and “Dark Turquoise” module (**D**) revealed the hub genes colored by red.

KEGG pathway enrichment analysis highlighted those genes in four modules (Black, Dark Turquoise, Brown, and Red) were involved in key pathways, including “MAPK signaling pathway—plant”, “Plant hormone signal transduction”, “Plant–pathogen interaction”, “Phenylalanine metabolism”, “Phenylpropanoid biosynthesis”, and “Transcription factors” (Table 1). Gene interaction network analysis using Cytoscape 3.6.1 identified 11 hub genes (Figure 4 and Table 2), with key candidates, such as *Bol035542* (dirigent protein, *DIR7*), *Bol043451* (jasmonate ZIM domain protein, *JAZ10*), *Bol010786* (Calmodulin binding protein, *CNGC13*), *Bol036026* (arogenate dehydratase 5, *ADT5*), *Bol017921* (G-type lectin S-receptor-like serine/threonine-protein kinase, *SD2-5*), and *Bol042792* (cytochrome P450, *CYP71B20*), selected for further in-depth study.

### 2.5. Preliminary Validation of Candidate Gene’s Function

**Figure 5:**
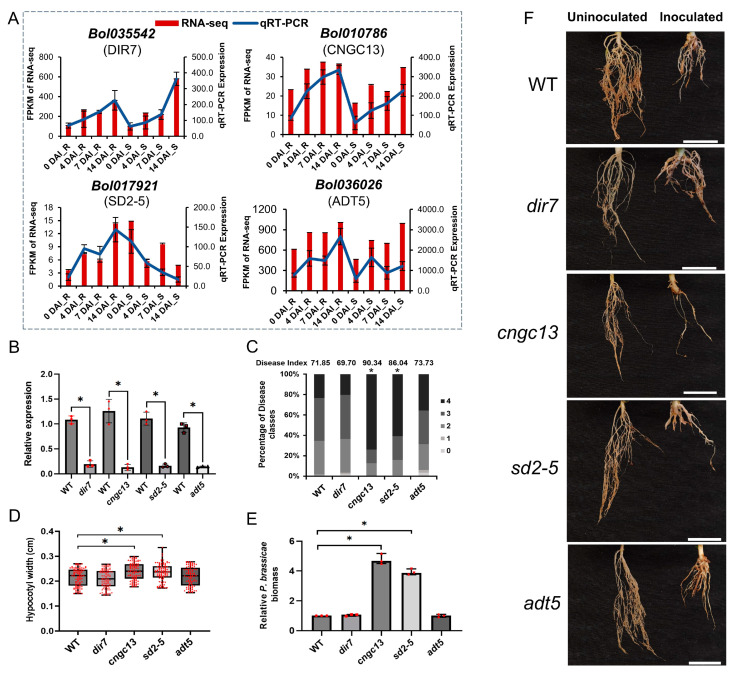
Validation of candidate genes through qRT-PCR and identification of clubroot resistance. (**A**) The expression levels of the four genes measured by RNA sequencing and qRT-PCR. (**B**) Relative gene expression in WT and T-DNA mutant lines of four candidate genes in *A. thaliana* at 28 DAI by *P. brassicae*. (**C**) Disease index and percentages of WT and four mutant lines in the individual disease classes. Disease index = (1 × n1 + 2 × n2 + 3 × n3 + 4 × n4) × 100/4Nt, where n1–4 represents the number of plants in each severity class, and the total number of plants tested is denoted as Nt. (**D**) Hypocotyl width of WT and four mutants at 28 DAI by *P. brassicae.* (**E**) The *P. brassicae* biomass of root among WT and four mutants at 28 DAI by *P. brassicae.* (**F**) Root symptoms of WT and four mutants at 28 days after uninoculated and inoculated by *P. brassicae*. Treatments were replicated three times with 34–45 plants per replicate. White bar: 1 cm. The asterisk indicates a significant difference at *p*-value ≤ 0.01.

The four genes (*Bol035542*, *Bol010786*, *Bol017921*, and *Bol036026*) were chosen for initial functional validation in clubroot. The RNA-Seq results of these four genes were validated by qRT-PCR (Figure 5A); then, we purchased the T-DNA mutants of their homologous *Arabidopsis* genes (*dir7*, *cngc13*, *sd2-5* and *adt5*).

The qRT-PCR results demonstrated that the four mutant plants exhibited lower expression levels compared to the WT (Figure 5B). Assessments of resistance to clubroot were conducted by measuring disease incidence, disease index, hypocotyl width, and *P. brassicae* biomass. The *cngc13* and *sd2-5* mutants showed more severe clubroot symptoms (Figure 5F). Compared to the WT, the disease index increased significantly by 25.73% and 19.75%, respectively (Figure 5C). Both the hypocotyl width (Figure 5D) and *P. brassicae* biomass (Figure 5E) were also significantly greater. In contrast, *dir7* and *adt5* did not exhibit significant changes in CR compared to the WT. These results suggest that the *CGNC13* and *SD2-5* genes play a role in conferring resistance against clubroot, highlighting the potential research significance of *Bol010786* and *Bol017921* in developing clubroot-resistant varieties.

## 3. Discussion

*P. brassicae* is a biotrophic pathogen that has a complex life cycle, including resting spores, primary infection, and secondary infection. Moreover, the exact timing of the life cycle remains poorly understood [3,4], and how *B. oleracea* resistance defends against *P. brassicae* is ambiguous.

In our study, we used the R pool and S pool constructed in the laboratory to perform transcriptome sequencing at different inoculation periods and performed heat map analysis on the expression changes in all DEGs (Figure 1B). We found that the R pool and S pool had different differences before and after inoculation. Expression profiles illustrate different responses of the two pools to *P. brassicae* infection. Based on the results of GO and KEGG enrichment analysis, it can be inferred that the R pool primarily generated a substantial amount of reactive oxygen species (ROS) through the ETI response upon infection by *P. brassicae*. This response involved the induction of numerous R genes that recognized the effector of the pathogen and participated in signal transduction. Additionally, a significant number of Cytochrome P450 were inducted in the R pool, while a considerable number of peroxidase genes were repressed. On the other hand, the S pool appeared to exhibit a slightly faster response against *P. brassicae*, primarily through the PTI response. This response involves the induction of a variety of genes, encoding calcium-binding proteins, calcium-dependent protein kinases, JAZs, WRKY TFs, MYB TFs, and peroxidase. In parallel, there was an up-regulation of numerous genes related to carbon source and nitrogen metabolism. However, there was a suppression of genes involved in auxin and cytokinin signal transduction in the S pool during 14DAI, which might have been associated with clubroot formation.

### 3.1. R Proteins Play Important Roles in R Pool Resistance to P. brassicae by ETI

The secreted effectors of the pathogen could suppress host resistance processes in favor of colonization [23,24,25], and the host possesses internal immune receptor-disease resistance genes (R genes) that encode NB-LRR proteins, which recognize effectors and trigger defense responses [26,27]. But only a few R genes, like *CRa* [13], *CRb* [15], and *Crr1a* [14], have been cloned and conferred race-specific resistance. Nevertheless, the effectiveness of these resistance genes has been undermined by newly evolved isolates of *P. brassicae*, including PbXm, posing a threat to their future use in crop protection [28,29]. In our study, many NB-LRR genes were mainly activated in the R pool, including four new genes, and a stronger ETI response could be revealed at the cortical infection stage compared with the root-hair infection stage (Supplementary Figure S3). Among these R genes, *Bol037412* exhibited high expression levels in black rot-resistant genotypes during resistance studies [30]; it was highly homologous with the Arabidopsis gene *AT5G17680.1* and may be associated with soybean rust (ASR) disease resistance [31]. *Bol_new_8994* is a homolog of *TAO1* that contributes to the target AvrB effector of *P. syringae* [32]. The two R genes were induced to up-regulate in the R pool compared to the S pool, which requires further investigation.

### 3.2. PTl Contributes to Effective Resistance to P. brassicae in S Pool

PTI response is basic resistance for plants by interactions between PRRs of the host and PAMPs of the pathogen [33] and coexistence with ETI response to promote host resistance to pathogens [11]. Based on the results of GO and KEGG enrichment analysis, it was determined that the disease resistance mechanism in S pool primarily involved the PTI response, and the chitinase, PRRs, MAPK, WRKYs, lignification, and Ca^2+^ signaling showed regulation in S pools compared to R pool (Supplementary Figure S3).

Chitin is an extensively studied PAMP, a major component of fungal cell walls, and it is also the main carbohydrate present in spore cell walls of *P. brassicae* [34]. Chitinases belong to the pathogenesis-related protein category and are induced in clubroot-susceptible genotypes upon infection with *P. brassicae* [18]; this is consistent with the variation in the three chitinase genes in the S pool in our results (Supplementary Figure S3). In the S pool, which contained 2 *CERK1*, *FLS2*, and 1 *BAK1*, the PPRs were found to be up-regulated. Additionally, genes associated with MAPK signaling were also up-regulated in the S pool. This is consistent with previous research, which demonstrates that chitin recognition by CERK1 triggers immune activation through a MAPK signaling cascade. Furthermore, PbChiB2, a protein from cabbage, has been shown to inhibit the activation of this crucial MAPK pathway for plants to escape PTI [34].

*WRKY*s and Ca^2+^ signaling play key roles in both PTI and ETI [35,36]. Previous studies have reported that in transcriptome analysis of cabbage inoculated with *P. brassicae*, 11 *WRKY*s were found to be up-regulated in R plants [18]. Furthermore, some studies have suggested that the distinct regulation of *WRKY* genes may have contributed to the activation of their respective defense pathway resistance to *S. sclerotiorum* [37], and several *WRKY*s acted as negative regulators of plant defense [38]. Calcium could also reduce root hair infections, and Ca^2+^ signaling was reported to be associated with the resistance in hosts to *P. brassicae* [39,40]. In our study, 17 WRKY transcription factors and 14 Ca^2+^ signaling genes were found to be both up-regulated and down-regulated in both the resistant (R) and susceptible (S) pools, suggesting that WRKY transcription factors and calcium signaling may play distinct roles in the response to *P. brassicae* in these pools. Additionally, pathways associated with lignin biosynthesis were up-regulated during root-hair infection by *P. brassicae*, which aligns with the observed up-regulation of lignification-related genes in the S pool in our study.

### 3.3. Plant Hormone Participate in Responses to P. brassicae

Plant hormones regulate many features that are important for the growth of the clubroot in *Brassica* species. CTK, auxin, and brassinosteroids (BRs) are the primary hormones that regulate the cell cycle [41], which is crucial for the growth of clubroot in plants [42]. After *P. brassicae* has established itself in the root cortex, cell enlargement and growth occur [43,44].

Auxin and BRs are responsible for both cell division and cell elongation in a developing plant [45]. In our study, BR-activated transcription factor-encoding genes (Brassinosteroid resistant 1/2, *BZR1/2*) and BR receptor gene (Brassinosteroid insensitive 1, *BRI1*) were found to be up-regulated, while *BRI1* kinase inhibitor 1 (*BKI1*) and Cyclin D3 (*CYCD3*) were down-regulated in the S pool compared with the R pool. Previous studies have shown that aberrantly expressed *BZR1*, *BRI1*, and *BKI1* could contribute to clubroot pathogenesis [46]. Additionally, plants with mutations in the BRI1 receptor or those treated with a BR biosynthesis inhibitor exhibit reduced gall size [42]. This suggests that targeting and inhibiting the aberrant expression of genes associated with BR biosynthesis may confer resistance against *P. brassicae* infection.

Additionally, in the R pool, two AUX/IAA genes, two GH3 genes, and two SAUR genes were up-regulated. Conversely, in the S pool, three AUX/IAA genes and four GH3 genes were up-regulated, while six SAUR genes were down-regulated (Supplementary Figure S3). Many genes involved in maintaining the balance of auxin levels have been found to have different expression patterns between R and S genotypes in various transcriptomic studies [16,19]. Up-regulation of *AUX/IAA*, *GH3,* and *SAUR* genes may aid in reducing the amount of IAA and, thus, alleviate the enlargement and expansion of root cells in the resistant genotype [47]. Our results suggest that the up-regulation of two *SAUR* genes in the R pool may play a role in regulating auxin levels, potentially mitigating the development of clubroot.

Similarly, three *CKX* genes and two *ARR* genes were up-regulated, and one *ARR* gene was down-regulated in the S pool, while almost all cytokinin-related genes were down-regulated in the R pool (Supplementary Figure S3). The findings of Malinowski et al. [48] suggest that up-regulation of the *CKX* gene can help alleviate clubroot. Similarly, Wei et al. [49] found increased up-regulation of ARR genes in S plants. In our results, both *CKX* and *ARR* were found to be up-regulated in the S pool, indicating that maintaining CKT (cytokinin) homeostasis might be advantageous in mitigating clubroot. The down-regulation of the *Bol045724* (*CKX6*) homolog in the R pool aligns with the study conducted by Ciaghi et al. [50]. However, further research is needed to elucidate the role of this gene in CTK homeostasis within the R pool.

It is widely acknowledged that jasmonic acid plays a role in the resistance of plants to biotrophic pathogens, including *P. brassicae* [51]. In the present study, 14 *JAZ* genes, one *JAR1* gene, and one *MYC2* gene were mainly activated in the S pool. There is evidence that JA is involved in disease resistance at the initial and later stages of *P. brassicae* infection [49,52], and the expression of JAZ is up-regulated, leading to the repression of JA signaling [53]. Hence, according to our research, the activation of the JA signaling pathway could potentially enhance the defense against *P. brassicae* JAZ.

### 3.4. The Hub Genes Involved in Resistance to P. brassicae

In our study, *Bol043451* (*JAZ10*) and *Bol042792* (*CYP71B20*) were down-regulated at 7 DAI and then up-regulated at 14 DAI in S pool, but *Bol043451* showed a consistent downward trend in R pool at 4, 7, and 14 DAI, while *Bol042792* only up-regulated at 14 DAI in R pool. JAZ proteins, including JAZ10, act as negative regulators in JA signaling [53], whereas CYPs are involved in the JA and methyl jasmonate (MeJA) signaling pathways [54] and mediate catabolism of the plant hormone jasmonoyl-L-isoleucine (JA-Ile), such as *CYP94B3* [55]. JAZ10 can be used as a reporter to screen for mutants of JA signaling in *Arabidopsis thaliana* [56], and alternative splicing of JAZ10 pre-messenger RNA creates a regulatory circuit to attenuate JA responses [57]. JA plays a role in disease resistance at both the early and late stages of *P. brassicae* infection [49]. The manipulation of these genes, particularly in enhancing SA signaling, could offer new avenues for breeding CR varieties.

*Bol010786* (*CNGC13*) was up-regulated after being inoculated with *P. brassicae*, but the R pool was extremely up-regulated at 14 DAI. Calcium could reduce root hair infections as well as inhibit the production of differentiated sporangia of *P. brassicae* [58]. Cyclic nucleotide-gated channels (CNGC) mediate Ca^2+^ influx into the cytosol following activation via ligand binding in plants [59]. *AtCNGC10* influences numerous growth responses and starch accumulation in *A. thaliana* [60]. Ning, Wang, Fang, Zhuang, Zhang, Lv, Liu, Li, and Yang [47] found that Bol010786 was up-regulated at a late stage of *P. brassicae* inoculation, which is consistent with our results. Our identification results also confirm the involvement of *Arabidopsis CNGC13* in clubroot resistance.

The *Bol036026* (*ADT5*) exhibited an overall higher FPKM level with an up-regulation trend in both the R and S pools, though it was slightly down-regulated at 7 DAI. Genes encoding arogenate dehydratase 4 and 5 (*ADT4* and *ADT5*, respectively) are used for phenylalanine synthesis and the active role of phenylalanine and its secondary metabolites in ETI [61]. This suggests that *ADT5* might influence disease resistance in the R pool, warranting further investigation.

The expression pattern of the *Bol035542* (*DIR7*) gene in the R and S pools was the same, and both were slightly down-regulated at 7 DAI, and the FPKM in the S pool was higher than that in the R pool. Many dirigent genes are inducible by different types of abiotic and biotic stress factors [62] and significantly up-regulated in response to fungal infection [63]. Expression of transcripts belonging to the dirigent-like protein family was most divergent between R and S plants at 14DAI, which is consistent with our results. Additionally, the down-regulation observed at 7 DAI in the S pool might have contributed to the progression of infection.

*Bol017921* (G-type lectin S-receptor-kinase, *SD2-5*) overall trend was up-regulated in the R pool and down-regulated in the S pool. L-type lectin receptor kinases (LecRKs) are a class of RLKs that serve as PRRs in recognizing stress signals and initiating plant defenses. Forty-five LecRK genes have been found in *A. thaliana*, several of which showed inducible expression during pathogen challenge in response to pathogen-associated elicitors and MAMPs [64,65]. But so far, no G-type lectin S-receptor-kinase has been found to play a role in plant disease resistance. However, our findings confirm the role of *Arabidopsis SD2-5* in clubroot disease.

Based on our subsequent analysis of clubroot resistance in *Arabidopsis* homologous mutants, *Bol017921* and *Bol010786* have been identified as particularly promising candidates for further investigation.

## 4. Materials and Methods

### 4.1. Plant Materials and P. brassicae Inoculation

On the basics of our previous studies [22], extreme R and S pools derived from the F2 segregating population developed by “GZ87” (resistant to *P. brassicae* race 4) and “263” (susceptible to *P. brassicae* race 4) were used in the present study. Each pool contained 20 extreme vegetative–propagated lines [66]. Vegetative plants were transplanted into 72-well plug trays after rooting and cultivated in a lighted incubator (16/8 h light/dark cycle under 25 ± 2 °C). Each plant was inoculated with 5 mL *P. brassicae* (race 4) resting spores suspension (4 × 10^7^ spores/mL) one week after transplanting [22,67].

### 4.2. Sampling, RNA Extraction, and Transcriptome Sequencing

According to the dynamics of the early stage of root infection by *P. brassicae* [3,4], root samples of each vegetative line were collected at 0 (no infection), 4 (root-hair infection), 7 (peak of primary infection and beginning of second infection), and 14 (peak of cortical infection) DAI, respectively, and then mixed into a resistant S pool at each time point, according to their resistance level. Three biological replicates were conducted, resulting in 24 root samples. Total RNA was extracted from each sample using TRNzol-A + Reagent (TianGen, Beijing, China), according to the manufacturer’s instructions. The RNA integrity, purity, and concentrations were assessed on the Agilent Bioanalyzer 2100 system. The cDNA libraries of 24 samples were generated and sequenced on an Illumina Hiseq 2000^TM^ platform, which was managed by the Biomarker Technologies Company in Beijing, China.

### 4.3. RNA-Seq Data Analysis and Gene Annotation

Based on our previous studies [22], the RNA-Seq data from the extreme R and S pools derived from the F2 segregating population developed by “GZ87” (resistant to *P. brassicae* race 4) and “263” (susceptible to *P. brassicae* race 4) were used in the present study. Before assembly, all raw data were quality-checked using FastQC v0.11.9, and then, the Illumina adapter was removed using Trimmomatic v0.36 [68]. Low-quality reads (>50% bases with quality scores ≤ 5) and reads containing ploy-N were removed from each raw data to generate clean data. The high-quality clean reads from all 24 samples were compared to *B. oleracea* genome (http://39.100.233.196:82/download_genome/Brassica_Genome_data/Braol_JZS_V1.1, accessed on 25 February 2021) [69] using Hisat2 software v2.2.0 [70]. Using the selected reference genome sequence, the mapped reads were spliced with StringTie v2.1.0 [71] software, and the original genome annotation information was compared to identify previously unannotated transcription regions. The discovery of new transcripts and genes in the species was facilitated by this process. Subsequently, the uniquely mapped reads were employed to calculate the relative abundance of genes using the FPKM (Fragments per kilobase per million) method [72]. DEGs were obtained using R package DESeq2 v1.44.0 [73] with default settings. The threshold determining the significance of DEGs among multiple samples used a false discovery rate (FDR) [74]. Genes with *FDR* ≤ 0.01 and |log_2_ (fold change)| ≥ 1 were considered as DEGs [75]. For convenience, DEGs that showed higher expression levels at 4, 7, and 14 DAI compared to 0 DAI were designated as up-regulated, while those with lower expression levels were designated as down-regulated.

All DEGs were functionally annotated using six databases, including the Cluster of Orthologous Groups of proteins (COG), Gene Ontology (GO), Kyoto Encyclopedia of Genes and Genomes (KEGG), Protein family (Pfam), Swiss-Prot, EggNOG [76] and NCBI non-redundant protein (Nr) databases, employing BlastX with an E-value < 1 × 10^−5^. The GO and KEGG enrichment analysis figures were generated using https://www.bioinformatics.com.cn (last accessed on 20 February 2023), an online platform for data analysis and visualization.

### 4.4. Gene Expression Validation

To validate the RNA-Seq results, quantitative reverse transcription PCR (qRT-PCR) was performed in triplicate using the CFX96 Real-Time PCR Detection System (Bio-Rad, Hercules, CA, USA), with 10-µL reaction systems. Each reaction contained 5 µL of 2 × SYBR Green Supermix (Bio-Rad, USA), 2.5 µL of first-strand cDNA synthesized from the same RNA samples mentioned above, 0.2 µL of forward primer, and 0.2 µL of reverse primer (10 µmol/L). The expression data were normalized to an internal control gene, *B. oleracea EF1α*. Data acquisition and analysis were performed using Bio-Rad CFX Manager™ Software v3.0, and the relative transcription level of each gene was estimated using the 2^−∆∆Ct^ method [77]. The gene-specific primers designed according to the gene sequences using Primer Premier 5 are listed in Supplementary Table S1.

### 4.5. Co-Expression Network Analysis and Prediction of Hub Genes

The co-expression gene network was constructed using the WGCNA package [78] in R software v3.1.0, following the tutorial on the WGCNA official website. Module identification was performed by merging modules with similar expression profiles at a merge cut height of 0.3. The interaction network of hub genes within each module was visualized using Cytoscape 3.6.1.

### 4.6. Preliminary Functional Verification of the Hub Gene

*A. thaliana* ecotype Columbia (Col-0) was used as the wild-type (WT) control in the present study. The *Arabidopsis* mutant *dir7* (SALK_046217C), *cngc13* (SALK_057742C), *sd2-5* (SALK_076637C), and *adt5* (SALK_088171C) were bought from the AraShare (https://www.arashare.cn/, accessed on 15 July 2022). The seeds were germinated in a growth chamber maintained at a temperature of 22/18 °C with a photoperiod of 16/8 h light/dark using a soil mixture of peat and vermiculite in a 3:1 ratio. All the mutant lines were further identified as a homozygous mutant using two gene-specific primers (LP and RP) and a T-DNA border primer (Supplementary Table S1).

For inoculation with *P. brassicae*, each plant was inoculated with 2 mL *P. brassicae* resting spores (1 × 10^7^ spores/mL) at three weeks of growth [22,67]. Disease severity was assessed at 28 DAI using a 0–4 scoring system modified from Siemens reported with three replications [79]. The score 0 indicated no galls; 1 indicated slight galls formation primarily on lateral roots; 2 indicated small galls on the main roots and multiple galls on the lateral roots; 3 indicated large and numerous galls on both the main and lateral roots, with few root hairs; 4 indicated severe galls on the lateral and main roots, with destruction or absence of the taproot. The disease index (DI) for each replicate was calculated using the individual severity ratings: DI = (1 × n1 + 2 × n2 + 3 × n3 + 4 × n4) × 100/4Nt, where n1–4 represents the number of plants in each severity class, and the total number of plants tested is denoted as Nt.

### 4.7. Quantification of P. brassicae Biomass

To quantify the biomass of *P. brassicae*, the below-ground parts of the infected root of *Arabidopsis* were harvested at 28 DAI. DNA was extracted using the cetyltrimethylammonium bromide (CTAB) method. *P. brassicae* biomass was determined by conducting a quantitative PCR analysis of the *P. brassicae* actin gene [80]. The *Arabidopsis actin* gene was used for normalization. The primers used for measuring *P. brassicae* biomass are listed in Supplementary Table S1.

## 5. Conclusions

In our study, we observed that a significant number of R genes in the R pool were induced to initiate ETI responses upon infection with *P. brassicae*. Meanwhile, candidate genes were screened using WGCNA, and two genes (*Bol010786* and *Bol017921*) were initially found to have the potential to confer resistance to clubroot.

Ultimately, this research could lead to the development of more robust, disease-resistant crop varieties, providing economic benefits and contributing to sustainable agriculture and global food security. Our findings advance the understanding of the mechanisms underlying CR and offer new avenues for research aimed at improving CR in *B.oleracea* through breeding programs.

## Figures and Tables

**Table 1:** KEGG enrichment analysis of modules significantly associated with R or S pools in WGCNA.

Module	Pathway	Description	Corrected *p*-Value	Associated Sample
Black	ko00400	Phenylalanine, tyrosine, and tryptophan biosynthesis	3.21 × 10^−4^	4 DAI_R, 14 DAI_R
	**ko00360**	**Phenylalanine metabolism**	**1.06 × 10^−3^**	
	**ko00940**	**Phenylpropanoid biosynthesis**	**1.16 × 10^−3^**	
	ko00710	Carbon fixation in photosynthetic organisms	1.29 × 10^−2^	
	ko02000	Transporters	2.70 × 10^−2^	
Brown	**ko04016**	**MAPK signaling pathway—plant**	**6.30 × 10^−7^**	4 DAI_R, 7 DAI_R
	**ko03000**	**Transcription factors**	**1.18 × 10^−5^**	
	**ko04075**	**Plant hormone signal transduction**	**1.97 × 10^−2^**	
	**ko04626**	**Plant–pathogen interaction**	**2.04 × 10^−2^**	
Dark magenta	ko03011	Ribosome	1.92 × 10^−13^	4 DAI_R
	ko00940	Phenylpropanoid biosynthesis	2.64 × 10^−6^	
	ko00040	Pentose and glucuronate interconversions	1.68 × 10^−5^	
	ko03032	DNA replication proteins	3.19 × 10^−3^	
	ko00001	Enzymes with EC numbers	9.22 × 10^−3^	
	ko03036	Chromosome and associated proteins	2.17 × 10^−2^	
Red	**ko03000**	**Transcription factors**	**4.95 × 10^−6^**	7 DAI_R
Tan	ko00001	Enzymes with EC numbers	1.03 × 10^−3^	14 DAI_R, 14 DAI_S
Dark turquoise	ko00001	Enzymes with EC numbers	6.61 × 10^−3^	4 DAI_R
	**ko00940**	**Phenylpropanoid biosynthesis**	**1.22 × 10^−2^**	
	**ko04626**	**Plant–pathogen interaction**	**1.81 × 10^−2^**	
Orangered4	ko02000	Transporters	1.22 × 10^−5^	7 DAI_S
	ko02010	ABC transporters	1.77 × 10^−5^	
	ko04090	CD molecules	4.53 × 10^−4^	
Light cyan	-	-	-	14 DAI_S

Note: The bold text indicates the KEGG enrichment pathways of interest to us. DAI, days after inoculation. R, resistant pool. S, susceptible pool.

**Table 2:** Detailed information statistics of the identified hub genes. DAI, days after inoculation. R, resistant pool. S, susceptible pool.

Gene ID in *B. oleracea*	Gene Names in *A. thaliana*	Annotation	KEGG Pathway	0DAI_R	4DAI_R	7DAI_R	14DAI_R	0DAI_S	4DAI_S	7DAI_S	14DAI_S
*Bol038571*	*CCR2*	Cinnamoyl-CoA reductase 2	Phenylpropanoid biosynthesis	243.84	387.78	399.57	439.84	138.95	380.41	259.43	780.15
*Bol035542*	*DIR7*	Dirigent protein	-	107.61	266.99	246.35	362.55	99.56	234.72	218.89	578.92
*Bol015013*	*HSFA2*	Heat stress transcription factor A-2	Transcription factors	3.24	3.10	4.12	21.02	3.74	3.00	5.17	10.33
*Bol040347*	*HIL1*	Triacylglycerol lipase	-	12.92	12.64	14.49	26.65	13.33	10.70	13.64	18.00
*Bol010786*	*CNGC13*	Calmodulin binding	Plant-pathogen interaction	23.13	33.71	37.32	36.43	16.26	25.58	22.08	34.61
*Bol036026*	*ADT5*	Prephenate dehydratase	Phenylalanine, tyrosine and tryptophan biosynthesis	611.41	860.92	855.71	1008.41	464.27	743.70	699.78	995.55
*Bol042792*	*CYP71B20*	Cytochrome P450	-	16.37	20.79	20.53	24.66	6.79	14.84	11.05	24.72
*Bol027538*	*NIA1*	Oxidoreductase FAD-binding domain	Nitrogen metabolism	204.24	218.43	249.16	285.27	124.94	175.22	174.72	298.13
*Bol042723*	*PI4K GAMMA 7*	Phosphatidylinositol 3- and 4-kinase	-	36.62	31.26	34.77	63.57	79.66	30.89	50.90	27.22
*Bol043451*	*JAZ10*	Jasmonate ZIM domain protein	Plant hormone signal transduction	173.12	201.30	208.97	108.56	78.24	219.79	118.89	239.36
*Bol017921*	*SD2-5*	G-type lectin S-receptor-like serine	-	3.59	7.29	5.92	14.33	14.84	5.69	9.57	4.68

Note: DAI, days after inoculation. R, resistant pool. S, susceptible pool.

## Data Availability

The data that support the findings of this study are available from the corresponding author upon request.

## Supplementary Materials

The following supporting information can be downloaded at https://www.mdpi.com/article/10.3390/ijms25179189/s1.
