# Supplementary material for: Comparative Analysis of Transcriptomes Reveals Pathways and Verifies Candidate Genes for Clubroot Resistance in Brassica oleracea
Source: Int J Mol Sci. 2024 Aug 24;25(17):9189. doi: 10.3390/ijms25179189 (PMC11395044; doi:10.3390/ijms25179189)
Supplement: Supplementary file 1 [file ijms-25-09189-s001.zip › Supplementary Materials Figures.pdf]

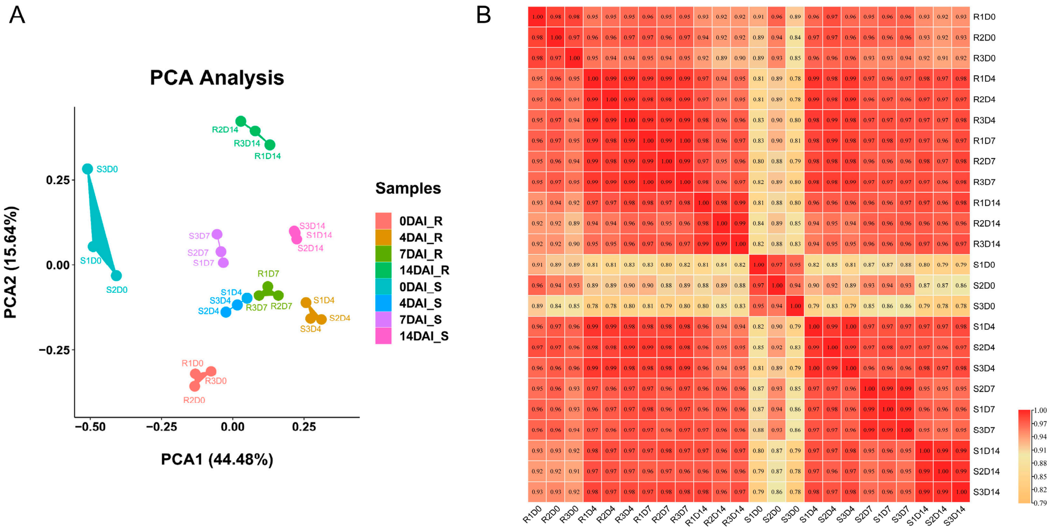

Figure S1 The relationship between transcriptome samples. (A) Principal Component Analysis (PCA) and (B) Pearson correlation were performed among three biological replicates at each infection stage. DAI, days after inoculation. R, resistant pool. S, susceptible pool.

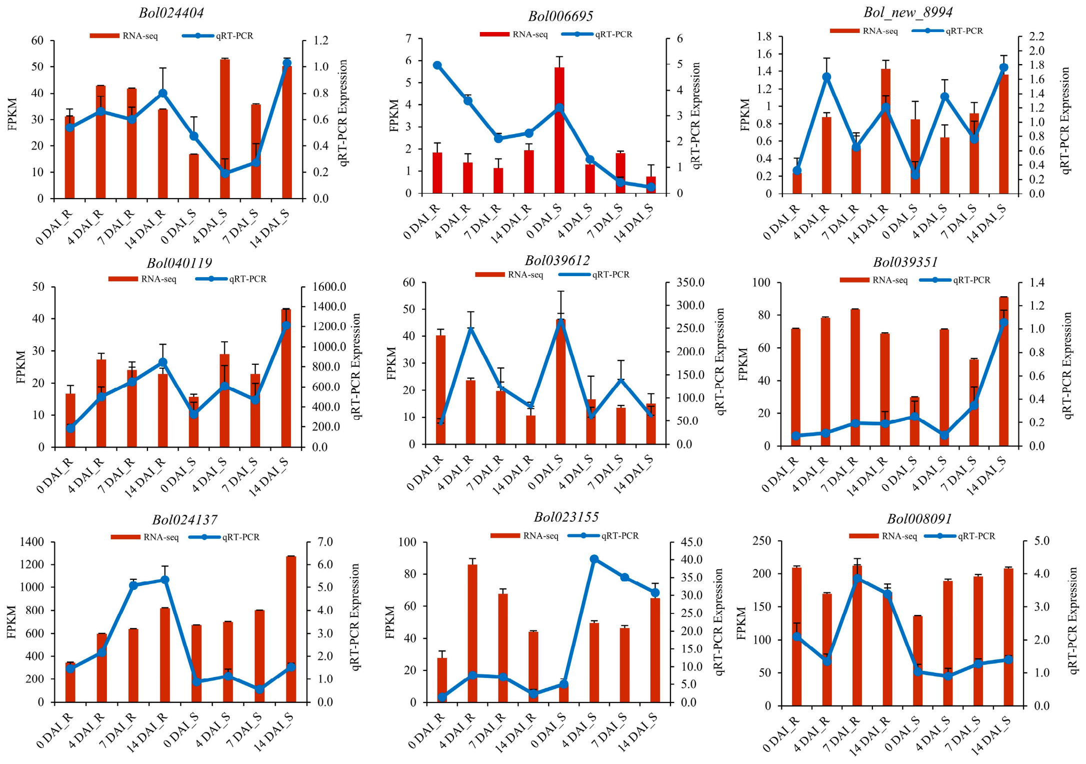

Figure S2 The expression levels of the nine selected genes measured by RNA sequencing and qRT-PCR. The data presented are the means of three replicates, and the error bars indicate the standard error. DAI, days after inoculation. R, resistant pool. S, susceptible pool.

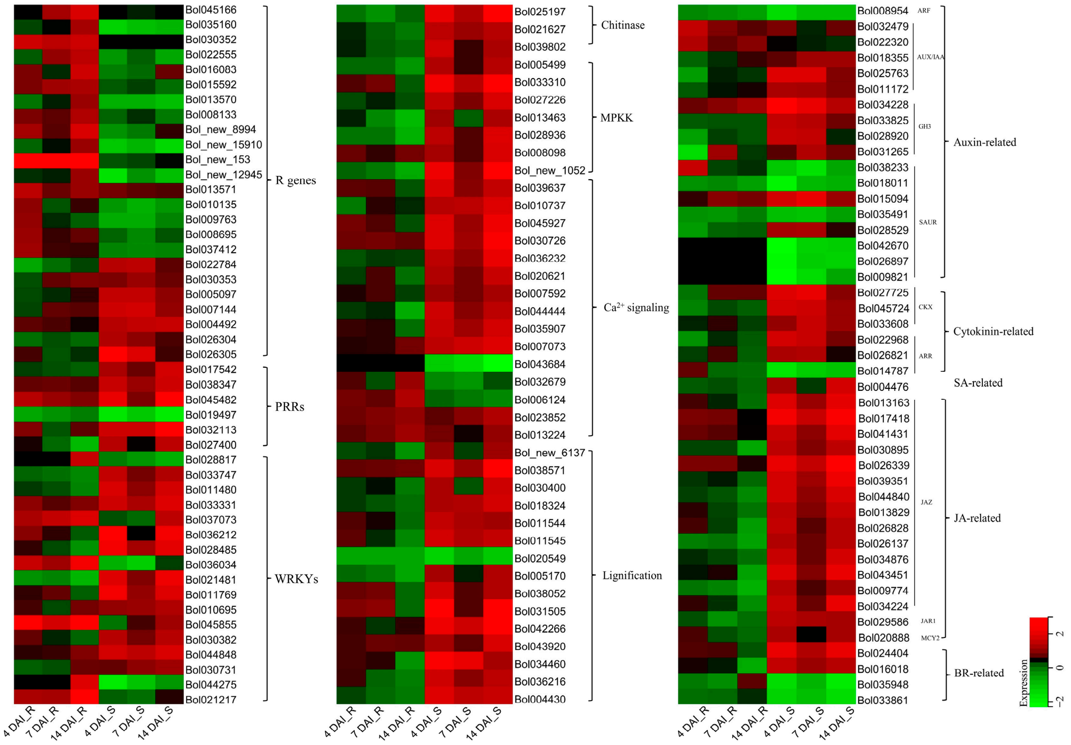

Figure S3 Heatmaps of DEGs responsive to *P. brassicae* infection in R and S pools at different infection stage compared to 0 DAI. The data presented are the means of three replicates, and be calculated based on the $\log_2$ (fold change) values. Green indicates down-regulated, and red indicates up-regulated. DAI, days after inoculation. R, resistant pool. S, susceptible pool.

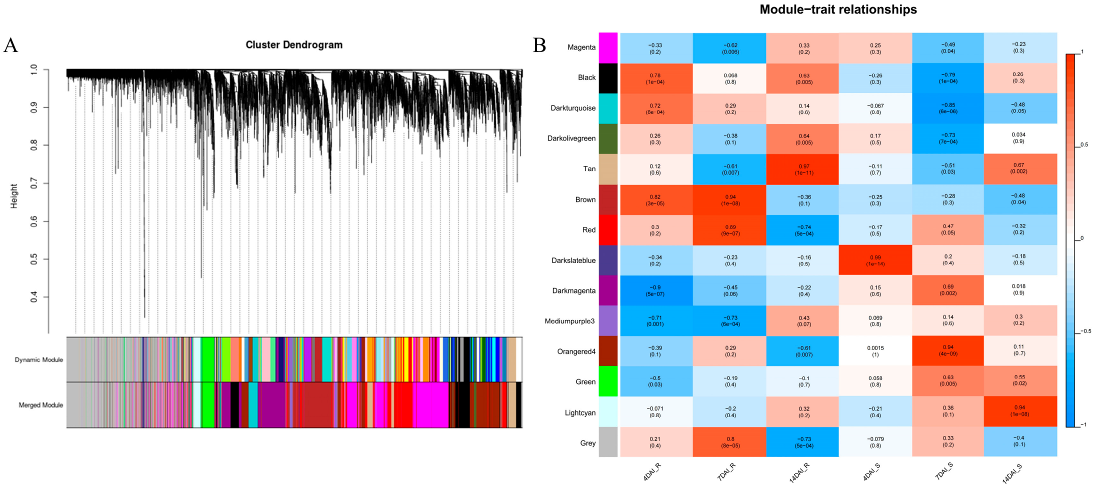

Figure S4 Weighted gene co-expression network analysis of DEGs at different infection stage compared to 0 DAI in R and S pools respectively. (**A**), Hierarchical cluster dendrogram showed co-expression modules. Modules, designated by color code, are the branches of the clustering tree. (**B**), Correlation analysis between module and *P. brassicae* infected samples. The number on each cell is the correlation coefficient between each module genes and infected sample, and the number below is the corresponding *p*-value. DAI, days after inoculation. R, resistant pool. S, susceptible pool.
